# Olanzapine-Induced Parkinsonism and Akathisia: A Case Report

**DOI:** 10.7759/cureus.21354

**Published:** 2022-01-18

**Authors:** Varun Jaitpal, Sushil Gawande

**Affiliations:** 1 Psychiatry, NKP Salve Institute of Medical Sciences and Research Centre, Nagpur, IND

**Keywords:** clinical case report, extrapyramidal side effects, akathisia, drug-induced-parkinsonism, olanzapine

## Abstract

This article sheds light on the case of a 22-year-old patient with paranoid schizophrenia. A treatment regimen of olanzapine (an atypical antipsychotic) of 15 mg/day was initiated for the patient and it was associated with extrapyramidal symptoms (EPS) such as drug-induced parkinsonism (DIP) and akathisia. Based on the different treatment regimens available, the patient was switched to an alternate antipsychotic along with anticholinergics, antihistamines, and benzodiazepines, following which the patient recovered well and did not report any further side effects. The highlighted case stresses the need for proper monitoring of the drug dose and sequential periodic examination of the patient to nullify the risk of adverse effects and poor compliance.

## Introduction

Nowadays, a multitude of treatment modalities, ranging from mono-drug therapy to combination therapies and interventions such as electroconvulsive therapy, are at a psychiatrist’s disposal for the management of patients suffering from a psychotic spectrum of diseases. Novel antipsychotic drugs such as risperidone and olanzapine have been used as the principal treatment modality to counter disorders of the psychotic spectrum [1]. Olanzapine, a second-generation antipsychotic also known as an atypical antipsychotic, was approved by the USA Food and Drug Administration (FDA) in 1996 for the treatment of schizophrenia [2].

A thienobenzodiazepine derivative, olanzapine is a second-generation antipsychotic, acting on dopamine, serotonin, histamine, and alpha-adrenergic receptors [3]. Along with all other antipsychotics available at present, olanzapine acts mainly by blocking D2 receptors [4,5]. This D2 receptor blocking leads to extrapyramidal symptoms (EPS) characterized by rigidity, parkinsonism, tremors, etc. Second-generation antipsychotics, including olanzapine, also block 5HT2A receptors, which help mediate EPS by potentially enhancing dopamine action [6].

Drug-induced parkinsonism and akathisia are potential complications of antipsychotic drugs and may significantly hamper the patients’ compliance with medication. Although olanzapine is considered to have a lesser propensity to cause extrapyramidal side effects and it is relatively prolactin-sparing [7], literature shows the association between the use of olanzapine and the manifestation of EPS is available.

Of all the movement disorders caused by agents acting on dopamine receptors, DIP is the most common [8]. The blockage of D2 receptors by antipsychotics in the striatum leads to disturbances in the basal ganglia-motor loop similar to the pathophysiology of Parkinson’s disease (PD) [8]. Therefore, the important features of DIP include tremors (symmetrical/asymmetrical) with bradykinesia and rigidity [8]. Akathisia is a subjective feeling wherein the patient feels restless with an intense urge to move [9]. The patient, however, is often unable to describe this discomfort and uses terms such as "anxiety" for its expression. Therefore, there is a lack of communication between the patient and the doctor. It may be "mildly annoying," or may even be "absolutely intolerable," leading to self-harming behavior [10]. This can be seen objectively as repetitive movements in the form of leg crossing, swinging, or pacing around [9]. The various risk factors leading to akathisia include high or rapid dose increase, trauma to the brain, deficiency of micronutrients like iron, etc. The differential diagnosis for akathisia includes anxiety, agitation with a medical condition, movement disorders like tics, and tardive dyskinesia [10].

This article aims to demonstrate the occurrence of olanzapine-associated DIP and akathisia with a case report. It also highlights the need for periodic and systematic examination of the patient to counter adverse drug reactions. All the necessary measures have been taken to ensure the confidentiality of the patient.

## Case presentation

A 22-year-old male patient presented with his brother to the emergency room of our hospital with chief complaints of restlessness, an intolerable urge to pace around, suspiciousness, and sleep disturbances in the form of late-onset and midnight awakenings for four days. Upon eliciting a detailed history, it was established that a couple of weeks ago, he was taken to the outpatient department (OPD) of a private psychiatrist with a history of suspiciousness, disorganized behavior in the form of muttering to self and poor self-care, withdrawn behavior, and sleep disturbances in the form of late-onset and midnight awakenings since one month. His brother reported a change in the patient’s behavior in the past few months, being suspicious and withdrawn as opposed to his premorbid extrovert personality. The patients’ heredity was not burdened by mental disorders. There was a history of daily cannabis consumption by the patient, with the last consumption being over two years ago, along with occasional consumption of alcohol and tobacco chewing, not following a dependence pattern. At initial presentation, the patient denied any illness, which was indicative of poor insight. After an adequate assessment, the patient was diagnosed with schizophrenia, with multiple episodes currently in an acute episode as per the Diagnostic and Statistical Manual of Mental Disorders, Fifth Edition (DSM-5) diagnostic criteria. The patient had been prescribed antipsychotics in the past but was off medication at the time of presentation at the private OPD. He was started on olanzapine at 2.5 mg/day on the first day by a private psychiatrist. This was subsequently increased to 5 mg/day on day 3. At this juncture, the patient reported episodes of mild restlessness, which did not interfere with the patient’s daily activities but caused discomfort. To counter this, propranolol 20 mg/day was added on day 3. Olanzapine was further increased to 15 mg/day on day 5 to relieve psychotic symptoms. Six days after maintaining the dose at 15 mg/day, he developed extrapyramidal symptoms in the form of tremors, slowness of movement, decreased arm swing while walking, and mask-like faces. This was followed by subjective reporting of severe restlessness along with objective evidence of akathisia since day 10. On day 14, the patient was referred to the hospital due to the gradual worsening of the above complaints and was admitted for further management. Physical examination of the patient at the time of admission revealed a positive glabellar tap sign. A diagnosis of drug-induced parkinsonism (DIP) and akathisia was made. For the disabling akathisia, the patient was continued on propranolol 20 mg/day and clonazepam 1 mg/day was added, while trihexyphenidyl 3 mg/day (2 mg tablet as 1-1/2) was added to treat the DIP. Olanzapine was serially withdrawn at the rate of 5 mg every two days and was omitted completely by day 4. Risperidone 2 mg was started on admission as the patient had received risperidone in the past with a good response and minimal side effects. On day 2 of admission, trihexyphenidyl was increased to 4 mg/day (2 mg tablet as 1-1/2-1/2). Even after seven days of continuous administration of the above regimen, the akathisia and DIP did not subside. Therefore, propranolol was increased to 40 mg/day and promethazine (Phenergan) at 25 mg/day was added on day 8. Risperidone was increased to 4 mg/day on day 4 to control psychotic symptoms. This was followed by increasing it to 6 mg/day on day 8 and later to 8 mg/day on day 12. Risperidone was given in equally divided doses twice daily. After one week (day 15 of admission), the akathisia and DIP completely subsided, and he was maintained on risperidone at 8 mg/day. Clonazepam was reduced to 0.75 mg per day on day 28. Careful observation, interviews, and periodic mental status examinations revealed no DIP or akathisia but good clinical improvement in psychotic symptoms, and the patient was discharged on day 30 of admission.

## Discussion

As mentioned earlier, olanzapine blocks serotonin as well as dopamine receptors and becomes an effective antipsychotic only when 60-70% D2 blockade is achieved. This is seen at doses of around 10 mg per day. At higher doses, it tends to block 80% of D2 receptors, which leads to EPS [11]. Skyes et al. proved that not just affinity to the receptors but the dissociation (*k*_off_) and association (*k*_on_) kinetics of the drug to the receptor, coupled with other receptors like the 5HT group, led to antipsychotic-induced EPS [12]. Beta-adrenergic blockers like propranolol are widely prescribed as first-line drugs for drug-induced akathisia, whereas anticholinergics and benzodiazepines are the second and third-line drugs, respectively [13].

The rate of EPS associated with the use of olanzapine is comparatively low. About 25-30% of patients taking a dose of olanzapine over ≥7.5 mg reported EPS. However, when compared to a placebo, this difference reached statistical significance only at doses of 12.5 mg. Large doses of olanzapine in the form of long-acting injectables (LAI) can be administered. The regimens that are available for olanzapine LAI are 150 mg every two weeks, 300 mg every two weeks, and 405 mg every four weeks. Here, olanzapine attains a steady-state concentration by 12 weeks after the first dose. The dose is calculated as per the oral olanzapine dose being administered [14]. The oral drug and the LAI for olanzapine both have a similar safety profile, and the incidence of motor adverse reactions as well as weight gain is seen in both forms [15]. The rates for akathisia were lowest for olanzapine (10%) [7], which were similar to quetiapine (13%) [10]. However, a few articles state that clozapine has a lower probability of causing akathisia than others, while other articles report a prevalence as high as 30% [10].

This clinical presentation outlines how the patient developed DIP and akathisia upon starting an olanzapine regimen. The temporal relationship between the introduction of the olanzapine regimen and the occurrence of EPS with marked improvement in symptoms after withdrawal of the drug supports the hypothesis that olanzapine was the cause of EPS. Very few case reports highlighting this association has been published to date, and the present case report highlights the importance of systematic hiking of doses coupled with periodic examination and monitoring of the patient to avoid adverse drug reactions.

## Conclusions

From the above case report, it may be inferred that olanzapine may cause EPS in the form of DIP and akathisia in patients who receive higher doses. Clinical presentation and good observation and examination allow the attending clinician to establish the diagnosis and manage the patient. Prevention of EPS or diagnosis and treatment at the earliest will ensure adequate compliance by the patient, which in turn will improve the overall prognosis.
